# A Case of Progressive Locomotor Ataxy

**Published:** 1866-07

**Authors:** J. P. Ross

**Affiliations:** Attending Physician to the Cook County Hospital


*A Case of Progressive Locomotor Ataxy.* By J. P. Ross, M. D., Attending Physician to the Cook County Hospital.

(Reported to the Chicago Medical Society.)

John W——, aged 37, sailor, a native of England, was admitted into the City Hospital, February 16, 1860. He stated that he had enjoyed good health until three years previously. In the spring of 1857 he felt a prickling sensation, followed by a numbness in the toes of the left foot, which gradually extended over the foot and leg. This was attended by a diminution of voluntary power in the parts. His right leg became similarly affected.

From this date his power of walking became impaired. His general health was good, and he continued his work until May, 1858. At this time he sailed to the West Indies with a hope of obtaining benefit from a change of climate, but returned in no way improved. During the following winter, wandering pains were felt in the left leg, and soon the knee became painful, hot and swollen. Blisters were applied, and the mur. tinct. ferr. given internally.

In three weeks the left knee got well, and the right one became affected in the same way, the difficulty disappearing after the same length of time. At this date sensibility was very imperfect, and the power of using the lower extremities was almost entirely gone. A pain was felt between the scapulæ; counter-irritation was now applied to the spine, and strychnia given by the attending physician until involuntary startings of the legs occurred, but no improvement in the power of motion. Since this time there has been but slight improvement.

On admission, his general appearance was healthy. Muscular development of lower limbs good. Was able to draw up his legs in the bed, or to move them in any direction; but in attempting to stand, he experienced great difficulty in steadying his legs sufficiently to bear his weight upon them; succeeded, however, after great effort, by leaning much of his weight on the back of a chair. On attempting to walk—by sliding the chair before him—his legs were jerked outwards, and soon losing control of them, the patient would fall upon the floor. The sensibility of the feet and legs was much reduced; numbness was felt in the ends of the fingers of both hands; suffered at times with sharp, shooting pains through the body and limbs, like the pains of rheumatism; the urine dribbled away involuntarily; the bowels were moved with difficulty; generative power extinct; other functions normal.

The doctor remarked that this case was under observation before the discovery of Duchenne was known to the profession in this country, and although the history did not give the minutiæ of symptoms, it contained the principal features of the disease, of which the chief was the want of power of locomotion, which was not caused by muscular paralysis, but by a *loss of power to co-ordinate the muscular movements.*

The history revealed this patient able to move the lower limbs in every way when he lay on the bed or was seated on a chair, but he could not steady and regulate their movements so as to effect locomotion. This is the main feature of the disease. The sharp, shooting pains and the numbness of the extremities, presented by this case, are generally present in this disease, but may be absent. Of seven cases reported by Dr. Radcliffe, four of the number had not the pains, and two had no anæsthesia. And the symptoms presented by the rectum, bladder and generative apparatus generally found, as in the case reported, are not necessary to constitute the disease, the essential idea of which is expressed by the name—locomotor ataxy.

On the subject of treatment, the doctor had but little confidence in any method unless resorted to in the early stage of the disease. His case was kept in the hospital for about three months under the alternate influence of various remedies, among the chief of which were iron, phosphorus and cod liver oil, without any material benefit, and he was finally dismissed as incurable.

